# Identification of crucial genes involved in pathogenesis of regional weakening of the aortic wall

**DOI:** 10.1186/s41065-021-00200-1

**Published:** 2021-12-02

**Authors:** Hong Lin Zu, Hong Wei Liu, Hai Yang Wang

**Affiliations:** 1 Department of Vascular Interventional Surgery, the First Affiliated Hospital of Harbin Medical University, No.23 Youzheng Str, Nangang District, Harbin, 150001 Heilongjiang China; 2 Department of Vascular Surgery, Tian Jin First Center Hospital, Tianjin, 300192 China; 3 Department of Vascular Surgery, the Second Affiliated Hospital of Harbin Medical University, Harbin, 150086 Heilongjiang China

**Keywords:** Abdominal aortic aneurysm, Differentially expressed genes, Weighted gene coexpression network analysis, Crucial genes, Vascular smooth muscle cells

## Abstract

**Background:**

The diameter of the abdominal aortic aneurysm (AAA) is the most commonly used parameter for the prediction of occurrence of AAA rupture. However, the most vulnerable region of the aortic wall may be different from the most dilated region of AAA under pressure. The present study is the first to use weighted gene coexpression network analysis (WGCNA) to detect the coexpressed genes that result in regional weakening of the aortic wall.

**Methods:**

The GSE165470 raw microarray dataset was used in the present study. Differentially expressed genes (DEGs) were filtered using the “limma” R package. DEGs were assessed by Gene Ontology biological process (GO-BP) and Kyoto Encyclopedia of Genes and Genomes (KEGG) analyses. WGCNA was used to construct the coexpression networks in the samples with regional weakening of the AAA wall and in the control group to detect the gene modules. The hub genes were defined in the significant functional modules, and a hub differentially expressed gene (hDEG) coexpression network was constructed with the highest confidence based on protein–protein interactions (PPIs). Molecular compound detection (MCODE) was used to identify crucial genes in the hDEG coexpression network. Crucial genes in the hDEG coexpression network were validated using the GSE7084 and GSE57691 microarray gene expression datasets.

**Result:**

A total of 350 DEGs were identified, including 62 upregulated and 288 downregulated DEGs. The pathways were involved in immune responses, vascular smooth muscle contraction and cell–matrix adhesion of DEGs in the samples with regional weakening in AAA. Antiquewhite3 was the most significant module and was used to identify downregulated hDEGs based on the result of the most significant modules negatively related to the trait of weakened aneurysm walls. Seven crucial genes were identified and validated: *ACTG2, CALD1, LMOD1, MYH11, MYL9, MYLK,* and *TPM2*. These crucial genes were associated with the mechanisms of AAA progression.

**Conclusion:**

We identified crucial genes that may play a significant role in weakening of the AAA wall and may be potential targets for medical therapies and diagnostic biomarkers. Further studies are required to more comprehensively elucidate the functions of crucial genes in the pathogenesis of regional weakening in AAA.

**Supplementary Information:**

The online version contains supplementary material available at 10.1186/s41065-021-00200-1.

## Introduction

Ruptured abdominal aortic aneurysm (AAA) is an important complication caused by AAA, resulting in severe haemorrhagic shock, and is associated with a mortality rate as high as 81% [1]. A considerable part of patients with ruptured AAA (rAAA) die before admission to the hospital. Most patients with AAA are asymptomatic, and population-based screening studies have reported the prevalence rates of AAA ranging from 1.6% to 7.2% of the general population from 60 to 65 years of age or older [2]. Smoking, uncontrolled blood pressure, older age, female sex and aneurysm diameter are risk factors for AAA rupture. In current clinical practice, the diameter of the aneurysm is the most commonly used parameter for the prediction of occurrence of AAA rupture, and patients with larger diameter aneurysms are at the highest risk of aortic rupture. The annual rupture rate is approximately 1% in patients with aneurysms 40–49 mm in diameter and 11% for patients with aneurysms 50–59 mm in diameter [2]. Elective surgical treatment includes open surgical repair or endovascular aneurysm repair provided to the majority of diagnosed patients at low or acceptable surgical risk with large AAAs ≥ 55 mm in diameter in men and ≥ 50 mm in diameter in women [3]. Despite a possibility of a rupture, regular follow-up is preferentially recommended versus surgical intervention in the case of small AAAs [4]; thus, specific drug therapy to limit the growth or rupture of AAAs is unavailable.

Molecular and histopathological evaluation of tissue samples of abdominal aorta provides a comprehensive approach to identify broadly coordinated gene expression in biological pathways involved in rAAA pathobiology. E Choke et al. [5] used the whole transcriptome approach to confirm overexpression of the genes *IL-8, IL-6, PTGS2, PROK2* and *SELE* involved in the immune and inflammatory responses at the AAA rupture sites. Gäbel et al. [6] utilized aortic wall tissue specimens for investigation of differentially expressed genes (DEGs) and validated that *HILPDA, ANGPTL4, LOX, SRPX2, FCGBP, ADAMTS9, STC1, GFPT2, GAL3ST4* and *CCL4L1* were overexpressed candidate genes related to angiogenesis, adipogenesis, epithelial-mesenchymal transition and the HIF-1a pathway apparently pivotal for AAA progression towards rupture. Based on publicly available original datasets, Lei et al. [7] performed weighted gene coexpression network analysis (WGCNA) to identify DEGs and infiltrated immune cells, including naïve B cells and both resting and activated CD4^+^ memory T cells, which are significantly upregulated in ruptured AAA. WGCNA was used to explore the system-level functionality of the genes [8]. Previous studies used WGCNA with microarray data to identify the hub genes in AAA that may act as potential targets for medical therapy and as diagnostic biomarkers [9, 10].

Recently, Vivian et al. [11] suggested that the most dilated regions of AAA are not the most vulnerable regions under pressure and are ruptured due to the presence of a weakened region in the aortic wall. Forneris et al. [12] defined the regional aortic weakness (RAW) index as the sum of time-averaged wall shear stress, intraluminal thrombus and large strain scores for each patch of the aneurysm, and the RAW index values > 6 according to the ultimate tensile strength (UTS) values indicate a weak wall. This approach will assist in improving clinical decision-making in the management of aortic pathology. In the current study, WGCNA was constructed using the high RAW index and control samples to identify crucial genes in the constructed gene modules that were involved in regional weakening of the aortic wall. These genes may provide evidence for identification of biological mechanisms of pathogenesis in rAAA.

## Materials and methods

### Data collection

The raw data of the GSE165470 [12], GSE7084 [13] and GSE57691 microarrays were acquired from the Gene Expression Omnibus (GEO) database (https://www.ncbi.nlm.nih.gov/geo/); the data were uploaded by the University of Calgary, Wayne State University School of Medicine and James Cook University. We used publicly accessible data and did not conduct human or animal experiments; hence, Institutional Ethics Committee approval was not required.

### DEG screening and functional annotation analysis

The “Limma” R package was used to evaluate DEGs of the GEO chip data. The DEG cut-off criteria were set as *P* < 0.05 and |log2FC|> 2, and the “ggplot” package was used to generate the volcano map. Upregulated and downregulated DEGs were used for Gene Ontology biological process (GO-BP) and Kyoto Encyclopedia of Genes and Genomes (KEGG) analyses by using the Database for Annotation, Visualization and Integrated Discovery (DAVID) v6.8 (https://david.ncifcrf.gov/) [14]. The top 10 statistically significant GO-BP and KEGG pathway terms for DEGs were recorded. The levels of enriched genes ≥ 2 and *P* < 0.05 were considered statistically significant differences.

### Construction of a weighted gene coexpression network and hub gene screening

The “WGCNA” R package was applied to generate a coexpression network to explore the clustered functionality of the genes and identify the core regulatory genes that were biologically meaningful. All genes were involved in the analysis. The absolute value of the Pearson correlation was calculated, and the raw gene matrix was converted into a similarity expression matrix. A suitable soft threshold (β) was selected to construct a network that accounted for the scale-free characteristics, and the linear regression model fitting index R2 was quantified to assess the scale-free topology of the network. Then, the weighted adjacency matrix was generated and transformed into a topological overlap matrix (TOM). The TOM-based dissimilarity measure in average linkage hierarchical clustering was used for functional module detection. Similar dynamic modules were merged based on the height cut-off of the dendrogram at the minimum module of 30. Highly similar dynamic modules were further merged at a threshold of 0.2.

### Construction of the DEG coexpression network

Module membership and gene significance were calculated to validate significant functional modules related to clinical traits. The hub genes were defined as the genes with the module membership > 0.8 in significant functional modules. Subsequently, a list of DEGs was overlapped with a list of the hub genes to construct a coexpression network of the hubs of differentially expressed genes (hDEGs).

### Construction of a PPI network and identification of crucial genes

The Search Tool for the Retrieval of Interacting Genes (STRING) database version 11.0 (string-db.org) was used to search for protein–protein interactions (PPI) with the highest confidence (interaction score > 0.9) to determine the interconnections of hDEGs in the functional modules [15]. Molecular complex detection (MCODE), a plug-in of Cytoscape 3.7.1, was used to detect and visualize the hDEG clusters, and hDEGs in the most significant clusters were considered crucial genes. Finally, crucial genes were validated based on the mRNA expression levels in the GSE7084 and GSE57691 raw microarray datasets by comparison between the AAA and control groups.

### Statistical analysis

R 4.1 was used for data preprocessing, DEG screening and WGCNA. DAVID was used for functional annotation analysis, and the MCODE plug-in of Cytoscape v3.7.1 was utilized to identify crucial genes. The criteria for these bioinformatic analyses are described in the corresponding subsections. Unpaired Student’s *t*-test was used to analyse the mRNA expression levels and compare the AAA and control groups. Statistical analysis was performed using GraphPad Prism v.6.01. *P* < 0.05 was considered significant.

## Results

### Data characteristics

The GSE165470 datasets included a total of two groups of 10 AAA patch samples. According to the RAW index (RAW > 6), 6 samples and 4 samples were assigned to the disease group and control group, respectively. The samples of the disease group had regional weakening of the AAA wall tissues. The gene expression matrix was acquired by transforming the probe ID matrix annotated with gene symbols from the GPL23126 platform data. The GSE7084 and GSE57691 microarray gene expression datasets contained 69 AAA tissues, and 21 controls were only used for validation of crucial genes. (Table 1).

**Table 1:** Description of the 3 GSE microarray datasets

Series	Tissues	Disease	Control	platforms
GSE165470	Abdominal Aortic aneurysm patch	6	4	GPL23126
GSE7084	Abdominal Aorta	10	11	GPL2507
GSE57691	Abdominal Aorta	59	10	GPL10558

### DEG screening

**Fig. 1:**
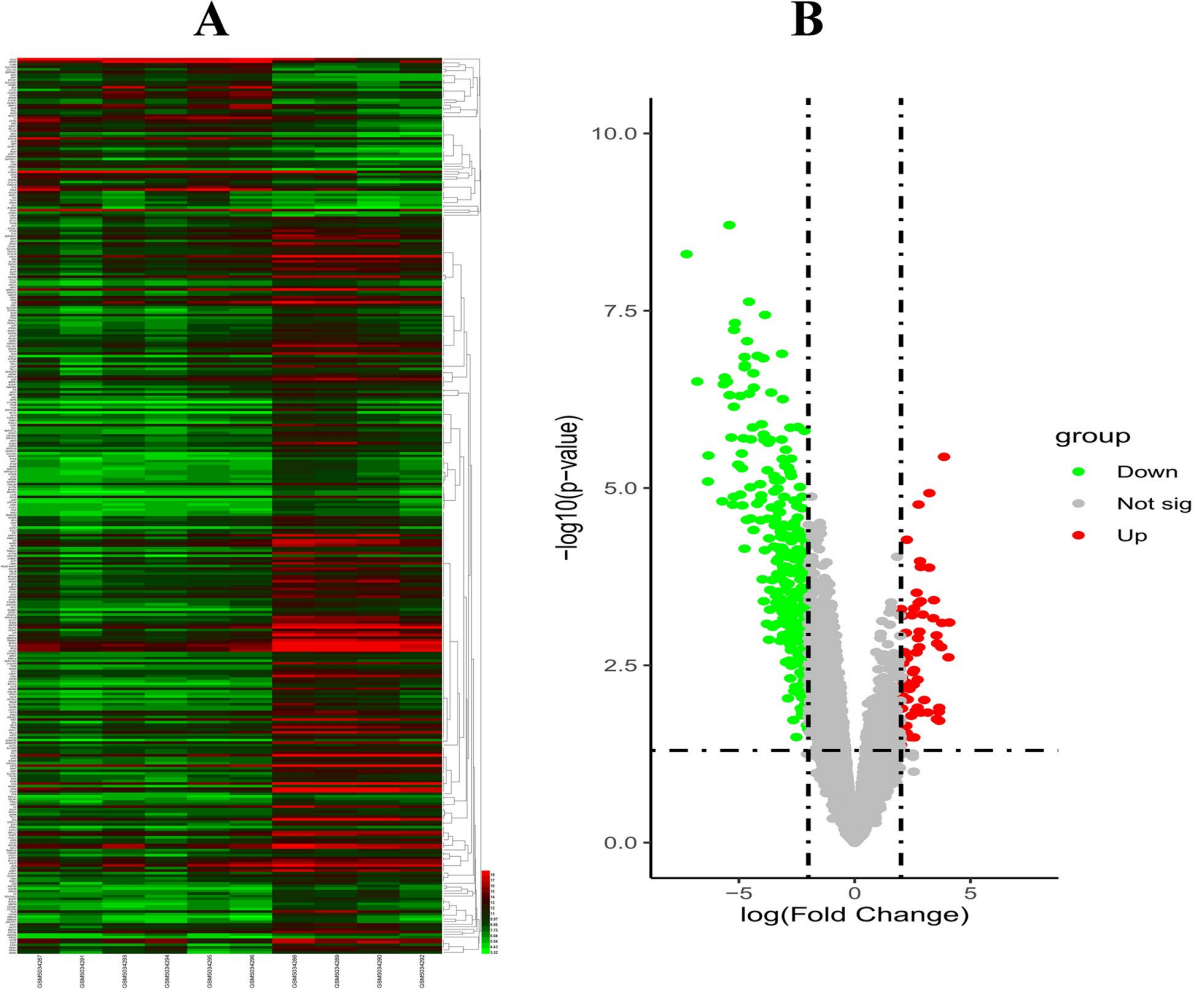
(**A**) Heatmap. The heatmap shows the expression levels of DEGs. (**B**) Volcano plot. The red dots represent upregulated DEGs, and the green dots represent downregulated DEGs

A total of 350 DEGs were screened with a *P*-value < 0.05 and a |log2-fold-change|> 2; these DEGs contained 62 upregulated and 288 downregulated genes. The expression levels and volcano plot of DEGs are visualized in Fig. 1A, B. The top 10 DEGs with the highest up- and downregulated expression levels are shown in Table 2.

**Table 2:** Top 10 up- and downregulated DEGs

Gene symbol	Log FC	*P*-Value	Gene official full name
UP-regulated			
*IGLL5*	4.085	0.000788	Immunoglobulin lambda like polypeptide 5
*SFRP4*	4.048333	0.00244	Secreted frizzled related protein 4
*SAA2*	3.856667	3.63E-06	Serum amyloid A2
*HLA-DRB5*	3.7725	0.000798	Major histocompatibility complex, class II, DR beta 5
*CD79A*	3.7375	0.00175	CD79a molecule
*IBSP*	3.65	0.019102	Integrin binding sialoprotein
*MMP12*	3.648333	0.012571	Matrix metallopeptidase 12
*CXCL13*	3.636667	0.014243	C-X-C motif chemokine ligand 13
*DCC*	3.555833	0.001552	DCC netrin 1 receptor
*FDCSP*	3.548333	0.018089	Follicular dendritic cell secreted protein
DOWN-regulated			
*CNN1*	-7.26083	5.03E-09	Calponin 1
*ITGA8*	-6.8025	3.15E-07	Integrin subunit alpha 8
*ACTC1*	-6.3375	8.10E-06	Actin, alpha, cardiac muscle 1
*MYH11*	-6.31667	3.48E-06	Myosin heavy chain 11
*MFAP4*	-5.71417	1.54E-05	Microfibrillar associated protein 4
*PLN*	-5.65833	3.44E-07	Phospholamban
*MYOCD*	-5.61333	2.76E-07	Myocardin
*PPP1R12B*	-5.51	3.17E-07	Protein phosphatase 1 regulatory subunit 12B
*RCAN2*	-5.41333	1.96E-09	Regulator of calcineurin 2
*PCDH7*	-5.405	4.90E-07	Protocadherin 7

### Functional annotation and pathway enrichment analysis

**Fig. 2:**
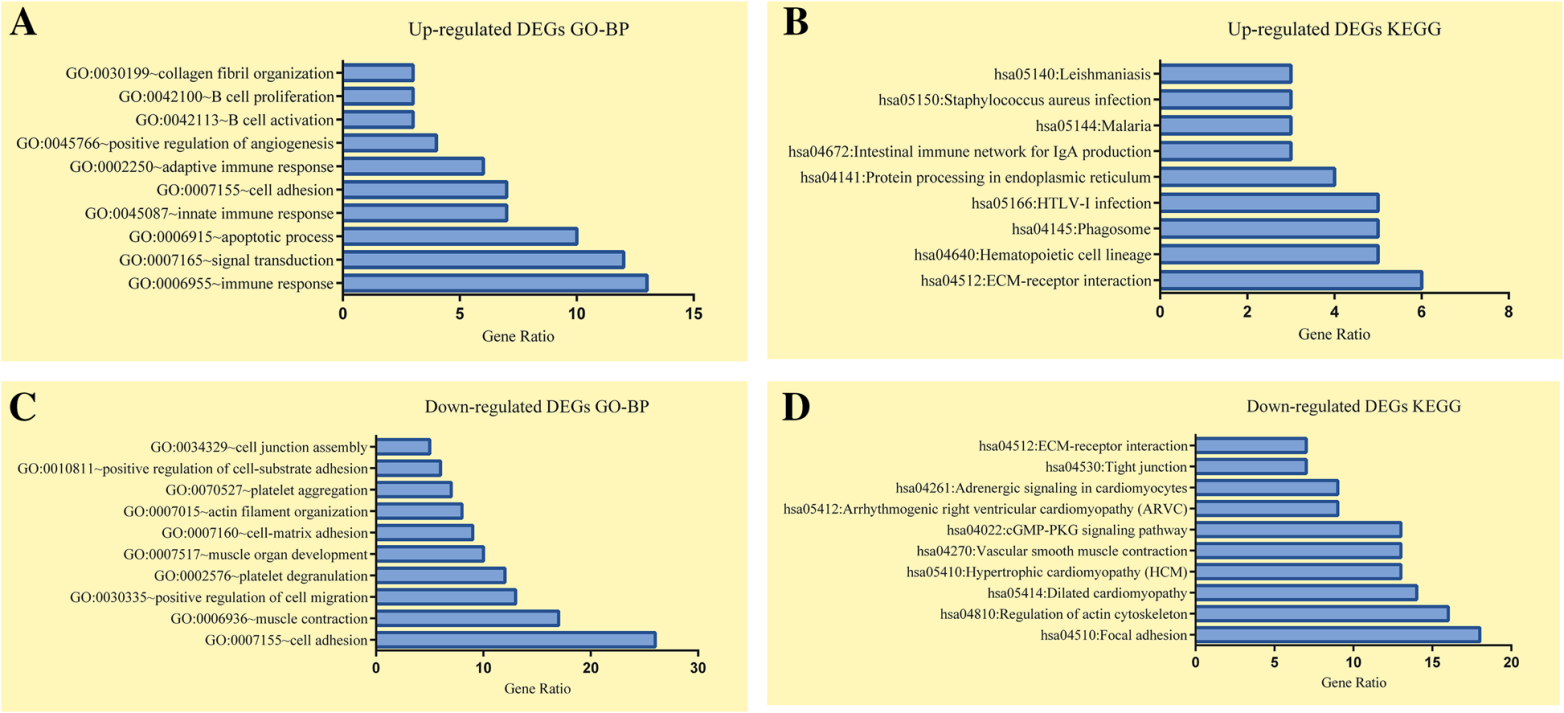
Top 10 GO-BP terms and KEGG pathways enriched in DEGs. (**A**, **B**) Functional enrichment analyses of the top 10 GO-BP and KEGG pathways enriched by upregulated DEGs according to the gene ratio values and in ascending order of the P-value. (**C**, **D**) The same criteria were applied for enrichment analysis of downregulated DEGs. The length of the histogram represents the number of the genes. Details of the GO-BP and KEGG pathway terms are described on the left Y-axis

A total of 35 GO-BP terms were enriched in upregulated DEGs, and 77 GO-BP terms were enriched in downregulated DEGs; the immune response and cell adhesion were the terms most significantly enriched by up- and downregulated DEGs in GO-BP. Nine and seventeen KEGG pathways were enriched in up- and downregulated DEGs, respectively, and ECM-receptor interaction and focal adhesion were the most significantly enriched KEGG pathways. The top 10 GO-BP terms and KEGG pathways enriched by DEGs are shown in Fig. 2, and DEGs involved in each enrichment pathway are shown in the supplementary file (Data S1).

### The results of weighted gene coexpression network analysis

**Fig. 3:**
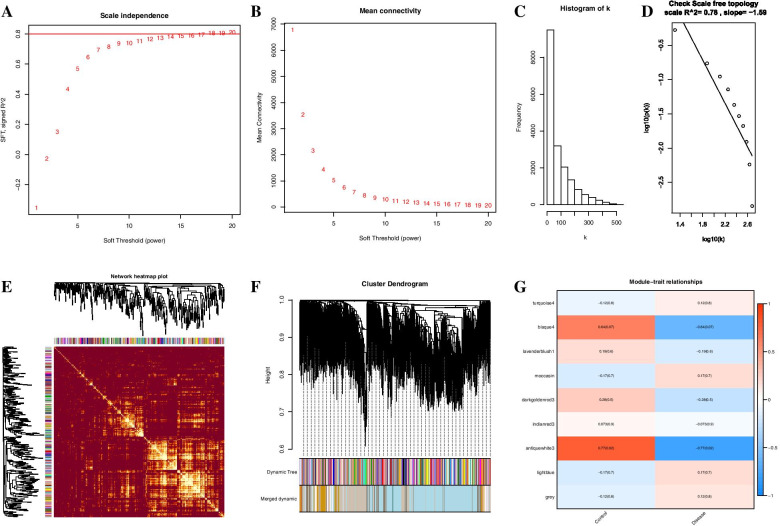
Algorithm of WGCNA. (**A**, **B**) An optimal soft-threshold power was selected to construct a scale-free topology network. (**C**, **D**) Scale-free topology with a soft threshold β value of 18. (**E**) Heatmap illustrating the TOM of the gene cluster tree; light colours represent a higher number of connections. (**F**) Module detection was based on hierarchical cluster analysis with cut-off and merging of the dendrogram, and the corresponding modules with various colours were obtained. (**G**) Calculated correlation coefficients between the modules and clinical traits. The antiquewhite3 module had the most negative correlation with the disease group

The “WGCNA” R package was used to construct a coexpression network for the 6 and 4 samples of the disease and control groups, respectively. The histogram and linear plot indicated that a cut-off R2 value of 0.78 and soft threshold β value of 18 should be used to build a coexpression scale-free topology network (Fig. 3A, B, C, D). Heatmap of the constructed gene coexpression network indicated the presence of several blocks with clear colour differences in the gene cluster tree (Fig. 3E). Nine highly similar dynamic modules were detected based on TOM matrices combined with cutting and merging of the dynamic dendrogram tree, and the antiquewhite3 module had the most significant negative correlation with the trait of weakened aneurysm walls (Fig. 3F, G).

### Identification of hub DEGs

**Fig. 4:**
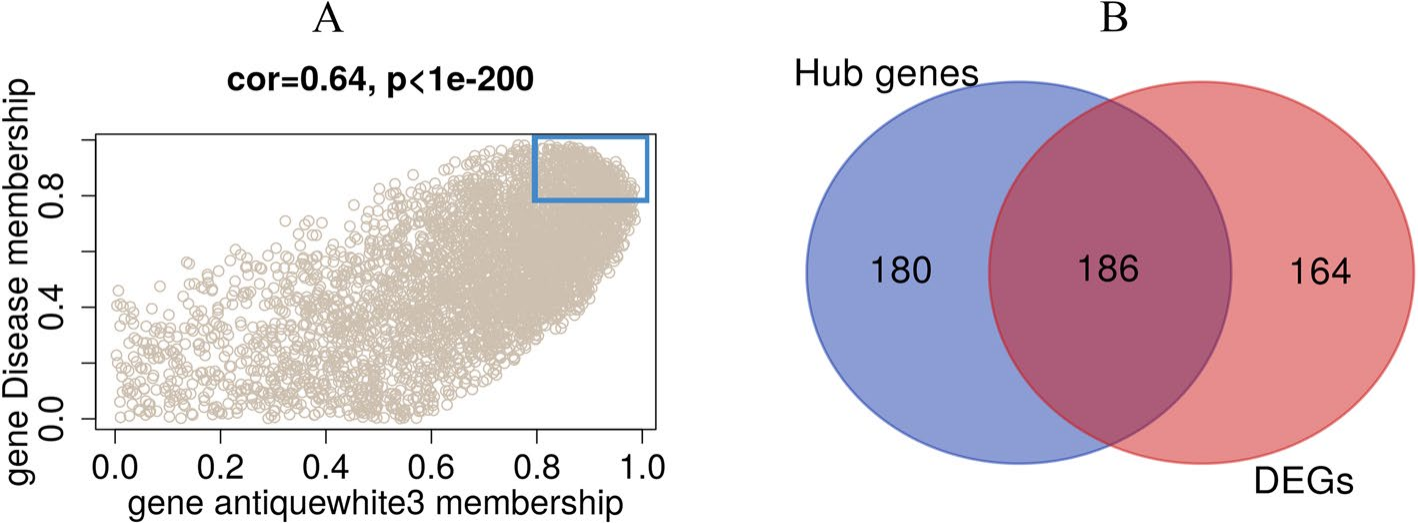
Algorithm of identification of the hub DEGs. (**A**) Gene sets highly related to the antiquewhite3 module and disease trait with a Pearson correlation coefficient of 0.64 and *P* < 0.05. Hub genes were identified with module membership > 0.8 in the blue rectangle. (**B**) A total of 186 downregulated hDEGs were obtained by overlapping 367 hub genes and 350 DEGs

The antiquewhite3 module included 3,912 genes (Fig. 4A). The 366 hub genes were identified by calculating the correlations to the antiquewhite3 module and the trait of weakened aneurysm walls. Then, the hub genes were overlapped with DEGs, and 186 downregulated hDEGs were identified (Fig. 4B), which was in agreement with the results of the most significant module that was negatively related to the trait of weakened aneurysm walls.

### Construction of a hub DEG coexpression network and identification of crucial genes

**Fig. 5:**
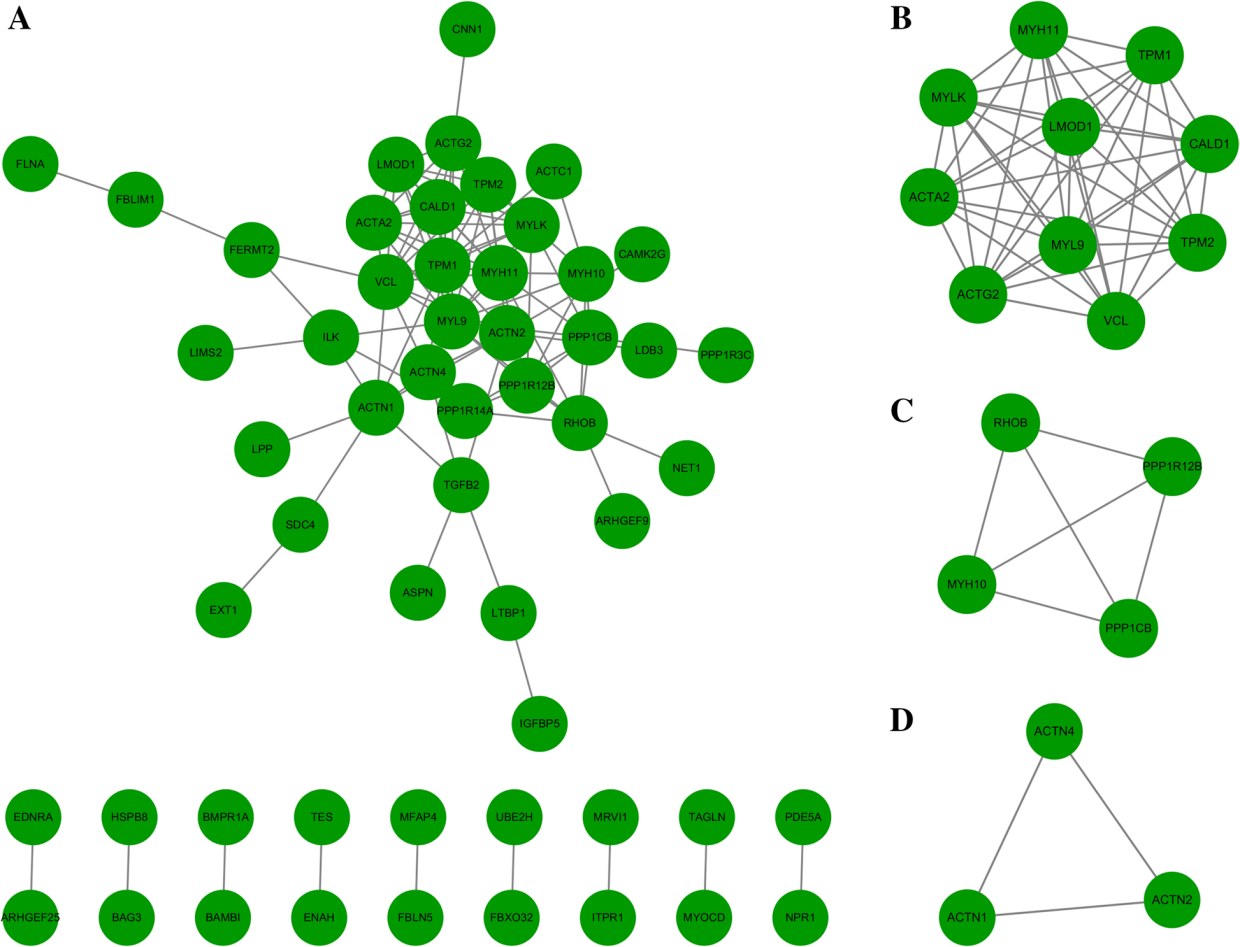
PPI networks of hDEGs and significant clusters. (**A**) An hDEG PPI network with 55 nodes and 109 edges. (**B**) Crucial genes were identified in the highest interconnected cluster. (**B**, **C**, **D**) Subnetwork of the interconnected hDEG clusters. The green dots represent downregulated genes

**Fig. 6:**
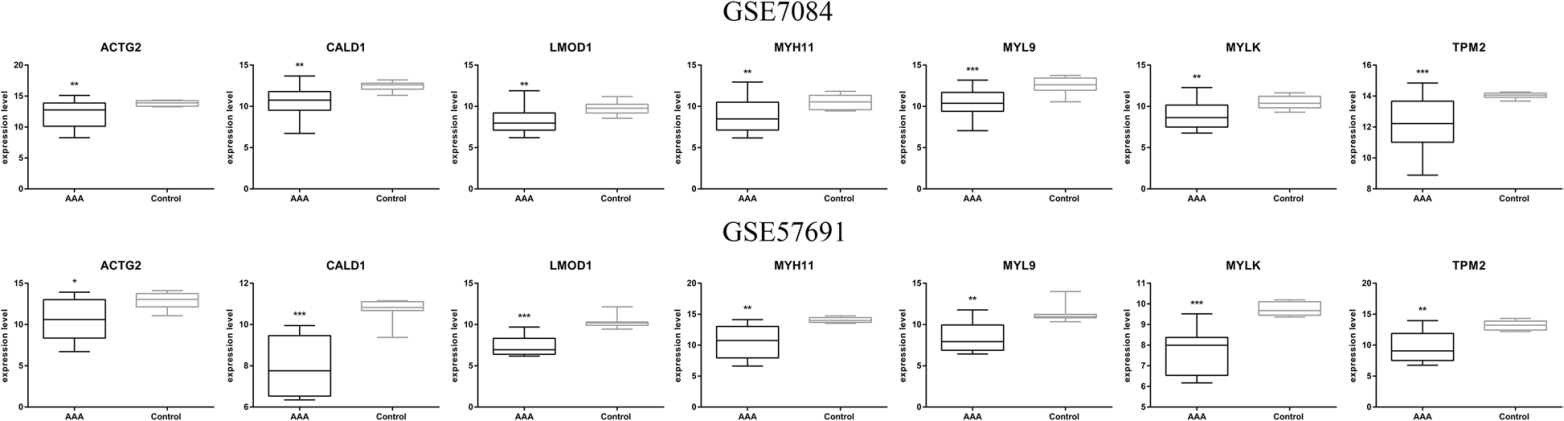
Validation of crucial genes. The crucial gene sets were validated using the mRNA expression levels of the GSE7084 and GSE57691 microarray (*P < 0.05)

All hDEGs were the genes downregulated in the samples with weakened regional aneurysm wall versus the samples of the control group. It is unlikely that a single gene or pathway can cause regional weakening of the aneurysm walls. An hDEG PPI network with 55 nodes and 109 edges was constructed with the highest confidence (Fig. 5A). In addition, 3 interconnected cluster networks of hDEGs were identified and visualized by using MCODE (Fig. 5B, C, D). *ACTA2, ACTG2, CALD1, LMOD1, MYH11, MYL9, MYLK, TPM1, TPM2* and *VCL* were crucial genes with the highest interconnected cluster scores (Fig. 5B). Apart from *ACTA2, TPM1* and *VCL*, all other crucial genes in the hDEG coexpression network were successfully validated using the GSE7084 and GSE57691 microarray gene expression datasets (Fig. 6 and supplementary file Data S2).

## Discussion

Genes in patients with ruptured AAA were likely to be more actively involved in inflammation, proteolysis, pathways regulating VSMC lineages and ECM homeostasis. In addition to the activation of immune responses, the AAA wall is characterized by proteolytic fragmentation of the extracellular matrix (ECM) and phenotypic modulation or apoptosis of vascular smooth muscle cells (VSMCs), which result in a thin and degraded media, few VSMCs and elastic components of the aneurysm walls [16, 17].

We identified 350 DEGs, including 62 upregulated and 288 downregulated genes, using the raw microarray data of GSE165470. The results of GO-BP and KEGG pathway enrichment analyses indicated that most of upregulated DEGs were involved in innate and adaptive immune responses, B cell activation and proliferation, positive regulation of angiogenesis, etc. In agreement with the results reported in human and animal models, pathological examination of AAA samples detected a wide array of innate and adaptive immune cells, such as mast cells, macrophages, neutrophils, dendritic cells, B cells and T cells, which infiltrated the aortic wall [18]. Inflammation and immunological responses play an important role in the progression of AAA. *HLADRB5* encodes the heavy chain of human leukocyte antigen (HLA) II, which activates T helper cells to increase the inflammatory response. Marfan syndrome patients with progressive aortic root dilatation have increased expression of *HLA-DRB5*, which indicates an increase in the inflammatory response corresponding to progressive aortic disease [19]. HLA II genes (HLA-DRB1*15, HLA-DRB1*04 and HLA-DRB1*02) have been identified as the factors involved in AAA development [20]. *CXCL13* promotes the migration of B lymphocytes and was validated as a novel inflammation gene linked to AAA based on increased gene expression profiles [21]. Kevin et al. [22] demonstrated that the media and adventitia layers of AAA contain higher levels of CXCL13, which triggers the recruitment of B cells, resulting in a high presence of B cells in the adventitia layer. Simultaneously, phenotypic changes in VSMCs can act as lymphoid tissue organizer-like cells in inflammatory environment involved in human aortic lymphoid neogenesis and are subjected to persistent inflammation and immune responses.

Interestingly, most downregulated DEGs were involved in various biological pathways, such as vascular smooth muscle contraction, regulation of actin cytoskeleton, cell–matrix adhesion and ECM-receptor interaction. *ITGA8* was expressed at a high level in aortic SMC tissue of human origin with contractile phenotype; moreover, ectopic MYOCD expression resulted in a consistent increase in *ITGA8* mRNA levels in rat, mouse and human SMCs [23]. Moreover, *ITGA8* may be an assembly that regulates normal SMC differentiation, and decreased *IGTA8* expression leads to impaired VSMC contractility [24]. MFAP4 is an ECM protein expressed at a high level in human elastic tissues specifically localized in elastic fibres in the blood vessels [25]. Lindholt et al. [26] used immunohistochemistry to demonstrate a higher MFAP4 staining in healthy tissues than that in AAA tissues, and high levels of plasma MFAP4 are significantly negatively correlated with the aorta growth rate, resulting in decreased surgical repair in AAA.

However, monogenic disorders are rarely associated with AAA progression. The present study is the first to construct a coexpression network for both high and low RAW index samples by using the WGCNA algorithm to detect the coexpressed genes resulting in weakening of the regional aortic wall. Seven crucial genes, *ACTG2, CALD1, LMOD1, MYH11, MYL9, MYLK,* and *TPM2,* were identified and validated; moreover, these coexpressed genes may play an important role in regional weakening the aortic wall.

ACTG2 belong to the actin family and is highly conserved cytoskeletal proteins. *ACTG2* encodes a protein specific to visceral smooth muscle tissue [27]. *MYH11* encodes a smooth muscle myosin belonging to the myosin heavy chain family. Utako et al. [28] demonstrated that circulating plasma MYH11 levels were significantly higher in AAA. *MYLK* encodes myosin light chain kinase, which phosphorylates myosin regulatory light chains to facilitate myosin interaction with actin filaments to produce contractile activity, and diminished levels of MYL9 cause a failure in actomyosin-based contraction [29].

The major function of VSMC contractile force requires cyclic interactions between SMC a-actin and the β-myosin heavy chain encoded by *MYH11* [27]. MYH11 and MYLK gene mutation-induced switching of aortic SMCs from a contractile to a synthetic inflammatory phenotype is accompanied by secretion of abundant MMPs, inflammatory cytokines and chemokines and growth factors and by production of reactive oxygen species (ROS) and proteoglycans in the aortic adventitia to create a vicious cycle leading to the degradation of ECM and weakening of the aortic wall, resulting in aortic rupture [30–32]. Shalata et al. [33] reported that the *MYLK* Ala1491Ser mutation affected kinase activity and clinically presented with vascular aneurysm and dissection in thoracic and/or abdominal aorta and peripheral or visceral arteries.

The *LMOD1* and *CALD1* were implicated in differentiation-adapted phenotype of smooth muscle cells induced by serum response factor (SRF)/myocardin (MYOCD) transcription factors [34, 35]. Knockdown of *LMOD1* resulted in increased proliferation and migration and decreased cell contraction of human coronary artery SMCs [36], and *CALD1* was significantly expressed at low levels in VSMCs from carotid artery plaques, which were identified as critically involved in the development and progression of atherosclerosis [37]. *TPM2* encodes beta-tropomyosin, a subtype of tropomyosin that belongs to the family of actin filament-binding proteins [38]. Downregulation of TPM2 may lead to disorders in the formation and movement of VSMCs in the development of atherosclerosis [39].

In conclusion, hDEGs are involved in phenotypic changes and even apoptosis of VSMCs, and degraded ECM results in regional weakening of the aortic wall. Consequent biomechanical stress exerted on the wall dilates the aneurysm that ruptures when the AAA wall stress exceeds the tensile strength of the wall. We identified crucial genes that play a notable role in regional weakening of the AAA wall. The present study provides a reference for future studies, elucidating detailed biological mechanisms of crucial genes in pathogenesis of rAAA.

## Supplementary Information

**Additional file 1.**

**Additional file 2.**

## Data Availability

The GSE datasets are available in the GEO database (https://www.ncbi.nlm.nih.gov/geo/).

## Abbreviations

AAA: Abdominal aortic aneurysm
rAAA: Ruptured abdominal aortic aneurysm
RAW: Regional aortic weakness
UTS: Ultimate tensile strength
GEO: Gene Expression Omnibus
DEGs: Differentially expressed genes
WGCNA: Weighted gene coexpression network analysis
GO: Gene Ontology
BP: Biological process
KEGG: Kyoto Encyclopedia of Genes and Genomes
DAVID: Database for Annotation, Visualization and Integrated Discovery
TOM: Topological overlap matrix
STRING: Search Tool for the Retrieval of Interacting Genes
PPI: Protein–protein interaction
MCODE: Molecular Complex Detection
hDEGs: Hub differentially expressed genes
